# Female reproductive factors and risk of external causes of death among women: The Japan Public Health Center-based Prospective Study (JPHC Study)

**DOI:** 10.1038/s41598-019-50890-x

**Published:** 2019-10-04

**Authors:** Shiori Tanaka, Sarah K. Abe, Norie Sawada, Taiki Yamaji, Taichi Shimazu, Atsushi Goto, Motoki Iwasaki, Hiroyasu Iso, Tetsuya Mizoue, Manami Inoue, Shoichiro Tsugane

**Affiliations:** 1 Epidemiology and Prevention Group, Center for Public Health Sciences, National Cancer Center, 5-1-1 Tsukiji, Chuo-ku, Tokyo 104-0045 Japan; 2 Department of Global Health Policy, Graduate School of Medicine, The University of Tokyo, 7-3-1 Hongo, Bunkyo-ku, Tokyo 113-0033 Japan; 3 Public Health, Department of Social Medicine, Osaka University Graduate School of Medicine, 2-2 Yamadaoka, Suita, Osaka 565-0871 Japan; 4 Department of Epidemiology and Prevention, Center for Clinical Sciences, National Centre for Global Health and Medicine, 1-21-1 Toyama, Shinjuku-ku, Tokyo 162-8655 Japan

**Keywords:** Epidemiology, Risk factors

## Abstract

Although empirical data suggest a possible link between female reproductive events and risk of nonfatal accidents and suicidal behaviors, evidence to determine these effects on mortality is scarce. This study investigated the association between female reproductive factors and the risk of external causes of death among middle-aged Japanese women. We used a population-based cohort study consisting of 71 698 women residing in 11 public health center areas across Japan between 1990 and 1994. Multivariable-adjusted Cox proportional hazard regression models were used to estimate hazard ratios (HRs) of the risk of all external causes, suicide, and accidents according to female reproductive factors at the baseline survey. During 1 028 583 person-years of follow-up for 49 279 eligible subjects (average 20.9 years), we identified 328 deaths by all injuries. Among parous women, ever versus never breastfeeding [0.67 (95% CI: 0.49–0.92)] was associated with a decreased risk of all injuries. Risk of suicide was inversely associated with ever versus never parity [0.53 (95% CI: 0.32–0.88)]. A lower risk of death by accidents was seen in ever breastfeeding [0.63 (95% CI: 0.40–0.97)] compared to never breastfeeding. This study suggests that parity and breastfeeding are associated with reduced risk of death by all external causes, suicide and/or accidents among Japanese women.

## Introduction

Globally, more than five million people die each year as a result of external causes. These deaths have now become a major public health concern^1^. Japan has experienced a slightly decreasing suicide rate since its peak in 1998^2^. However, more than 20 000 people die annually by suicide, giving Japanese women the second highest suicide rate among OECD countries after Korea^3^.

Early epidemiological studies focused on potential associations between reproductive factors, and suicide behaviors and nonfatal accidents. In contrast, few studies examined external causes of death. The first report on a possible link between parity and suicide was Durkheim’s hypothesis in 1966, which suggested that parenthood rather than marriage per se was an important factor in protecting against suicide^4^. Reported or proposed protective factors for suicide include being pregnant^5,6^, having children^4,7^, ever or multi parity^8–12^, late age at first birth^8^, and never use of oral contraceptives (OCs)^13^. Similar results were also noted for deaths by accident^11,13–16^. However, these findings remain inconsistent, and a specific mechanism to explain these associations has not been provided, except with regard to parity^12,13,17,18^.

Suicide attempts and completion are significantly more frequent at times of low or rapid states of decline in endogenous sex steroids (estrogen and progesterone), such as peri-menopause^19^, the postpartum period^20^, and the premenstrual and menstrual phases of the cycle^21,22^. The complicated interplay among female sex steroids and the neuroregulatory system may link reproductive events to mental illness and suicidality^20,22,23^. Since female suicide completers are more likely to have a history of self-harm/suicide attempts^24^, even events that occurred long before, such as menarche, may be worth considering as risk factors of their lifetime suicidality. Women with a hypoestrogenic period such as menopause may be at increased likelihood of accidents due to a decrease in musculoskeletal^25^ and cognitive function^26,27^.

To date, no study has comprehensively investigated reproductive factors as potential markers for mortality risk of external causes. Here, we investigated the association between female reproductive factors and risk of all-cause and major causes of external deaths among middle-aged Japanese women.

## Methods and Materials

### Study population

**Figure 1:**
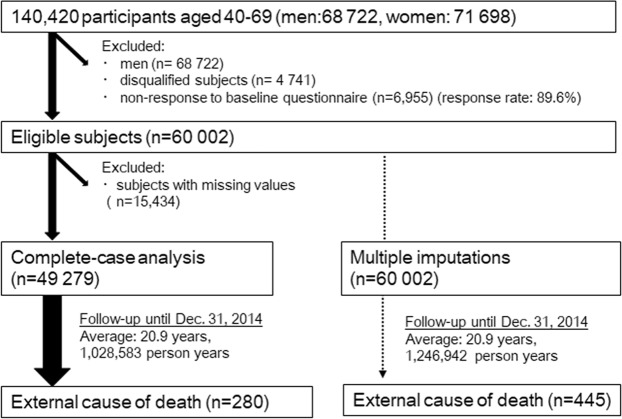
Study flow.

We used data of the Japan Public Health Center-based Prospective Study (JPHC Study), which is an ongoing population-based prospective cohort study. A total of 140 420 participants (68 722 men and 71 698 women) aged 40–69 in 11 public health center (PHC) areas nationwide enrolled between 1990 and 1994 (Fig. 1). The study areas were selected from across Japan based on geographical, environmental and cultural differences, and details of the JPHC Study have been described elsewhere^28–30^. A self-administered questionnaire was conducted at entry and at 5- and 10-year additional surveys to collect comprehensive information including lifestyle, personal and family medical history, diet and reproductive events. All methods described in the current study has been approved by the Institutional Review Board of the National Cancer Center (approval number: 2001–021) with reference to relevant ethical guidelines for medical research in Japan. Informed consent was obtained from all participants implicitly when completing the baseline questionnaire, in which the purpose and methods of study were well described and explained. The STROBE checklist was used to check items that should be included in the article.

Of the 71 698 women, those with non-Japanese nationality (n = 20), pre-commencement emigration (n = 86), incorrect birth data (n = 5), duplicate registration (n = 4) or late report of migration before the start of the follow-up period (n = 4 626) were excluded. Of those remaining, 60 002 women (89.6%) returned the completed questionnaire. Of eligible subjects, 49 279 (82.1%) completed relevant questions including parity, age at first birth, experience of breastfeeding, age at menarche, age at menopause, exogenous hormone use, height, weight, smoking habits, alcohol consumption, perceived stress level, living with a spouse, and history of disease.

### Follow-up and assessment of outcome

Participants were followed from the baseline survey (1990, 1994) until the date of death, last confirmed date of survival for participants who moved out of the study area (i.e., migration), or end of follow-up (December 31, 2014), whichever came first. The subjects from Katsushika and Suita public health center areas were scheduled to be followed for 20 years, until December 2009 and 2012, respectively. Survival or residential relocation of participants of the study areas was identified using public registries. During the study period, 8 477 (14.1%) died, 35 (<0.06%) emigrated outside of Japan, and 469 (0.8%) were lost to follow-up.

Death certificates were collected through the local public health centers and used to confirm the cause of death, with permission from the Ministry of Health, Labor and Welfare. Causes of death were defined according to the International Classification of Diseases, 10^th^ edition (ICD-10): all external causes (V01-Y89); intentional self-harm, namely suicide (X60-X84, Y87.0); and accidents (V01-X59, Y85-Y86).

### Exposure assessment

Reproductive events captured at the baseline survey were selected on the basis of prior research^31^, and categorized into tertiles or quartiles based on the frequency distribution within the cohort; the total number of live or still births (nulliparous versus parous, and 1, 2, 3, or ≥4 births), experience of breastfeeding (no or yes), age at first birth (≤23, 24–26, or ≥27 years), age at menarche (≤13, 14–15, or ≥16 years), exogenous hormone use (never or ever), menopausal status (pre-menopause, natural menopause, or surgical menopause) and age at menopause (pre-menopause, ≤47, 48–50, or ≥51 years). We calculated total fertility years as the interval between menarche and menopause (≤32, 33–36, or ≥37 years).

### Statistical analysis

All analyses were performed with STATA version 14.0 (StataCorp LP). Cox proportional hazards regression models were employed to estimate the hazard ratios (HR) and 95% confidence intervals (CI) to assess the risk of death by all external causes, suicide, and accidents according to reproductive factors using the STCOX command. Participants who were missing information on relevant reproductive factors or other covariates were excluded, leaving a total of 49 279 women in the primary analyses.

Age was used as the time scale for all models. The minimum model (Model 1) was built with stratification by 11 study areas to allow a different baseline hazard due to the varying distribution of suicide rates across Japan^32^. The second model (Model 2) was adjusted for a priori covariates and several reproductive factors as follows: body mass index (BMI, in kg/m^2^; <21.9, 22 to 24.9, or ≥25)^33,34^; smoking status (never or ever)^35^; alcohol consumption (no, occasional, or regular)^36^; perceived stress level (a little, average, or stressful)^37^; living with spouse (no or yes)^38,39^; past history of disease^35,40^, including cancer, stroke, heart disease, diabetes mellitus, and hypertension (no, or yes); parity^8^; age at menarche^14^; menopausal status^19^; and exogenous hormone use^13^. Living with a spouse was used as a surrogate of marital status. Breastfeeding and age at first birth^8^ were included in the second model when analyses were restricted to parous women.

Effects of *p*-values for linear trends were assessed for parity, age at first birth, age at menarche, age at menopause and total fertility years by assigning ordinal variables. A likelihood ratio test was conducted to compare models with and without interaction terms and to calculate a *p*-value for statistical interaction between reproductive factors and confounders. Proportional hazards assumptions were verified using Schoenfeld residuals, and no variable violated the PH assumption (Supplementary Fig. S1).

Stratified analysis by menopausal status at baseline was conducted because menopausal transition or menopausal status is likely to be a high risk for suicide or accidents^19,25–27^. For sensitivity analysis, we used the multiple imputations approach with 20 iterations to impute missing values to assess the degree of selection bias due to complete case analysis (details available in the footnote of Supplementary Table S2). The Fine and Gray model was conducted to assess competing risk of death from all causes, except external causes, using the STCRREG command^41^. All *p*-values reported were two-sided, and p < 0.05 was set as the significance level.

## Results

During 1 028 583 person-years (an average of 20.9 years) of follow-up for 49 279 women, a total of 328 deaths by all external causes, 148 suicides (45%), and 167 accidents (51%) were identified. In the remaining 4% of all external causes of death, we observed 6 (1.8%) deaths due to violence, 4 (1.2%) deaths due to an undetermined cause, and 3 (0.9%) deaths due to a medical procedure. The median age of death was 63 years old (IQR = 56–71) for suicide and 69 years old (IQR = 61–75) for accidents. In comparison with the age of deaths from all causes (73 years old, IQR = 65–80), study subjects died 10 earlier years by suicide.

Among subjects, 53.7% of women reported pre-menopausal status at the baseline survey (Table 1). Several variables varied by menopausal status; pre-menopausal women were younger, and reported less breastfeeding, younger age at menarche and more exogenous hormones use than post-menopausal women. When we compared the subjects with and without missing data for all relevant variables, 17.9% of subjects had at least one missing datum (Supplementary Table S1).

**Table 1:** Basic characteristics of study subjects at baseline survey of the JPHC study.

Characteristic	Total	Menopausal status at inclusion	
		Pre-menopause	Post-menopause
Number of subjects (n)	49 279	26 456 (53.7%)	22 824 (46.3%)
Age at recruitment, y^a^	50.9 (7.8)	44.6 (4.0)	56.3 (6.1)
BMI (Kg/m^2^)^a^	23.3 (3.2)	23.1 (3.1)	23.6 (3.3)
Never smoker (%)	90.3	88.2	83.1
Non-drinker (%)	74.2	66.5	80.8
High perceived stress (%)	16.5	23.2	16.5
Living with spouse (%)	79.2	82.7	76.2
History of diseases (%)	19.2	3.9	27.7
**Reproductive factors**			
Parity^a^	2.7 (1.5)	2.4 (1.2)	2.8 (1.7)
Age at first birth, y^a,b^	25.0 (3.5)	25.2 (3.5)	24.9 (3.5)
Ever breastfed (%)^b^	86.8	84.5	88.9
Age at menarche, y^a^	14.5 (1.9)	13.7 (1.5)	15.1 (1.9)
Age at menopause, y^a,c^	48.1 (4.8)		48.1 (4.8)
Total fertility years^a,c,d^	33.0 (4.8)		33.0 (4.8)
Ever use of exogenous hormones (%)	13.4	14.0	12.8

BMI, Body mass index; y, year.

^a^Mean (standard deviation).

^b^Parous women only.

^c^Post menopause only.

^d^Total fertility years as the interval between menarche and menopause.

Table 2 presents unadjusted and multivariable-adjusted HRs with 95% CIs of mortality risk by all external causes according to female reproductive factors for all women, with estimations for stratified analyses by menopausal status. A decreased risk of all external causes was observed in parous women with ever breastfeeding [0.67 (95% CI: 0.49–0.92)]. A marginally inverse association was found in women with three births compared to the reference group [2 births: reference; 1 birth: 1.07 (95% CI: 0.71–1.62); 3 births: 0.75 (95% CI: 0.55–1.01); ≥4 births: 0.99 (95% CI: 0.62–1.57): *P*_trend_ = 0.74]. A suggestive increased risk trend was found in women with later age at menarche [≤13 years: reference; 14–15: 1.48 (95% CI: 1.09–2.00); ≥16: 1.38 (95% CI: 0.97–1.96); *P*_trend_ = 0.07]. In stratified analysis, increased risk due to late age at menarche was more pronounced among pre-menopausal women. However, there was no statistically significant interaction among all reproductive factors.

**Table 2:** Hazard ratios (HRs) and 95% confidence intervals (CIs) of death by all external causes according to reproductive factors for all women, pre-menopausal women, and post-menopausal women in the JPHC study.

Variable	Category	Person-years	All women					Pre-menopause			Post-menopause			*P*_int_^c^
			Cases	Model 1^a^		Model 2^b^		Cases	Model 2^b^		Cases	Model 2^b^		
				HR	95% CI	HR	95% CI		HR	95% CI		HR	95% CI	
Parous	No	71 289	31	1.00	ref	1.00^h^	ref	13	1.00^h^	ref	18	1.00^h^	ref	0.18
	Yes	957 293	298	0.67	0.46–0.97	0.77	0.52–1.15	88	0.61	0.32–1.16	209	0.90	0.54–1.48	
Parity^d^	1	77 207	30	1.24	0.83–1.86	1.07	0.71–1.62	11	1.09	0.55–2.15	19	1.04	0.62–1.74	0.45
	2	371 068	117	1.00	ref	1.00	ref	45	1.00	ref	72	1.00	ref	
	3	291 703	71	0.74	0.55–0.99	0.75	0.55–1.01	17	0.53	0.55–2.15	54	0.86	0.60–1.24	
	≥4	217 314	79	1.06	0.76–1.48	0.99	0.62–1.57	15	0.94	0.49–1.80	64	1.12	0.75–1.65	
	*P*_trend_^g^			0.34		0.74			0.86			0.58		
Age at first birth, y^d^	≤22	213 668	76	1.00	ref	1.00	ref	21	1.00	ref	55	1.00	ref	0.89
	23–26	485 409	142	0.91	0.68–1.22	0.95	0.71–1.27	43	0.93	0.54–1.60	99	0.97	0.69–1.37	
	≥27	258 786	79	1.03	0.74–1.43	1.01	0.72–1.43	24	1.07	0.57–1.99	55	1.02	0.68–1.55	
	*P*_trend_^g^			0.84		0.94			0.82			0.91		
Breastfeeding^d^	Never	125 722	51	1.00	ref	1.00	ref	22	1.00	ref	29	1.00	ref	0.59
	Ever	831 570	246	0.64	0.47–0.88	0.67	0.49–0.92	66	0.62	0.38–1.02	180	0.71	0.47–1.08	
Age at menarche, y	≤13	316 896	65	1.00	ref	1.00	ref	27	1.00	ref	38	1.00	ref	0.12
	14–15	466 496	165	1.49	1.10–2.01	1.48	1.09–2.00	59	1.99	1.24–3.17	106	1.15	0.78–1.69	
	≥16	245 189	98	1.43	1.01–2.02	1.38	0.97–1.96	15	2.35	1.21–4.55	83	1.03	0.68–1.56	
	*P*_trend_^g^			0.06		0.07			0.01			0.88		
Exogenous hormone use	Never use	887 175	282	1.00	ref	1.00	ref	91	1.00	ref	191	1.00	ref	0.07
	Ever use	141 407	46	1.08	0.79–1.50	1.07	0.78–1.48	10	0.66	0.34–1.27	36	1.31	0.90–1.90	
Menopausal status	Pre menopause	481 912	101	0.94	0.62–1.44	1.00	0.72–1.37							
	Natural menopause	452 624	193	1.00	ref	1.00	ref							
	Surgical menopause	94 046	34	1.05	0.60–1.86	0.89	0.61–1.30							
Age at menopause, y^e^	≤47	177 875	72	1.00	ref	1.00	ref							
	48–50	210 330	86	0.95	0.69–1.31	0.92	0.65–1.30							
	≥51	158 485	69	0.94	0.66–1.32	0.92	0.63–1.33							
	*P*_trend_^g^			0.71		0.66								
Total fertility span, y^e,f^	≤32	204 419	82	1.00	ref	1.00	ref							
	33–35	167 339	70	0.97	0.70–1.34	0.94	0.67–1.33							
	≥36	174 911	75	0.95	0.69–1.31	0.92	0.64–1.33							
	*P*_trend_^g^			0.76		0.67								

HR, Hazard ratio; CI, Confidence interval; y, year; BMI, Body mass index.

^a^Cox proportional hazards models (using attained age as time scale) stratified by 11 public health center areas.

^b^Based on model 1 and adjusted for BMI, smoking habit, alcohol consumption, perceived stress level, living with a spouse, history of disease, parity, age at menarche, menopausal status, and exogenous hormone use.

^c^P value for interaction of likelihood ratio test.

^d^Parous women only with additional adjustment for age at first birth and breastfeeding.

^e^Menopausal women only.

^f^Total fertility years as the interval between menarche and menopause.

^g^*P* value for linear trend across categories of variable.

^h^Adjustments as in footnote b except for parity.

A lowered risk of suicide was evident in ever versus never parity [0.53 (95% CI: 0.32–0.88)] (Table 3). Parity with three births was inversely associated with risk of suicide compared to the reference group [2 births: reference; 1 birth: 1.12 (95% CI: 0.61–2.04); 3 births: 0.61 (95% CI: 0.39–0.97); ≥4 births: 0.91 (95% CI: 0.54–1.53); *P*_trend_ = 0.24].

**Table 3:** Hazard ratios (HRs) and 95% confidence intervals (CIs) of death by suicide according to reproductive factors for all women, pre-menopausal women, and post-menopausal women in the JPHC study.

Variable	Category	Person -years	All women					Pre-menopausal			Post-menopausal			*P*_int_^c^
			Cases	Model 1^a^		Model 2^b^		Cases	Model 2^b^		Cases	Model 2^b^		
				HR	95% CI	HR	95% CI		HR	95% CI		HR	95% CI	
Parous	No	71 289	20	1.00	ref	1.00^h^	ref	11	1.00^h^	ref	9	1.00 ^h^	ref	0.21
	Yes	957 293	128	0.45	0.28–0.73	0.53	0.32–0.88	54	0.47	0.23–0.94	72	0.64	0.30–1.36	
Parity^d^	1	77 207	14	1.23	0.68–2.20	1.12	0.61–2.04	6	0.96	0.40–2.34	8	1.21	0.55–2.65	0.33
	2	371 068	59	1.00	ref	1.00	ref	30	1.00	ref	29	1.00	ref	
	3	291 703	28	0.61	0.38–0.95	0.61	0.39–0.97	8	0.38	0.17–0.83	20	0.83	0.47–1.49	
	4+	217 314	27	0.95	0.58–1.59	0.91	0.54–1.53	10	0.96	0.44–2.11	17	0.95	0.49–1.85	
	*P*_trend_^g^			0.19		0.24			0.31			0.56		
Age at first birth, y^d^	≤22	213 668	32	1.00	ref	1.00	ref	14	1.00	ref	18	1.00	ref	0.72
	23–26	485 409	66	0.91	0.59–1.41	0.92	0.60–1.43	27	0.79	0.40–1.56	39	1.05	0.59–1.89	
	≥27	258 786	30	0.87	0.52–1.46	0.81	0.47–1.38	13	0.82	0.36–1.84	17	0.85	0.42–1.74	
	*P*_trend_^g^			0.61		0.44			0.63			0.66		
Breastfeeding ^d^	Never	125 722	23	1.00	ref	1.00	ref	14	1.00	ref	9	1.00	ref	0.32
	Ever	831 570	105	0.68	0.43–1.07	0.72	0.45–1.14	40	0.58	0.31–1.08	65	0.94	0.46–1.93	
Age at menarche, y	≤13	316 896	37	1.00	ref	1.00	ref	21	1.00	ref	16	1.00	ref	0.46
	14–15	466 496	75	1.36	0.91–2.05	1.36	0.90–2.05	35	1.54	0.89–2.68	40	1.09	0.60–2.00	
	≥16	245 189	36	1.42	0.86–2.34	1.37	0.82–2.28	9	2.08	0.92–4.70	27	0.98	0.51–1.89	
	*P*_trend_^g^			0.16		0.23			0.08			0.94		
Exogenous hormone use	Never use	887 175	129	1.00	ref	1.00	ref	59	1.00	ref	70	1.00	ref	0.28
	Ever use	141 407	19	0.88	0.54–1.44	0.85	0.52–1.40	6	0.64	0.27–1.51	13	1.02	0.55–1.89	
Menopausal status	Pre menopause	481 912	65	0.94	0.62–1.44	1.08	0.70–1.68							
	Natural menopause	452 624	68	1.00	ref	1.00	ref							
	Surgical menopause	94 046	15	1.05	0.60–1.86	0.99	0.56–1.77							
Age at menopause, y^e^	≤47	177 875	27	1.00	ref	1.00	ref							
	48–50	210 330	38	1.26	0.76–2.10	1.34	0.76–2.36							
	≥51	158 485	18	0.79	0.43–1.47	0.86	0.44–1.69							
	*P*_trend_^g^			0.52		0.64								
Total fertility span, y^e, f^	≤32	204 419	29	1.00	ref	1.00	ref							
	33–35	167 339	32	1.37	0.82–2.30	1.43	0.82–2.51							
	≥36	174 911	22	0.89	0.50–1.57	0.93	0.49–1.78							
	*P*_trend_^g^			0.73		0.82								

HR, Hazard ratio; CI, Confidence interval; y, year; BMI, Body mass index.

^a^Cox proportional hazards models (using attained age as time scale) stratified by 11 public health center areas.

^b^Based on model 1 and adjusted for BMI, smoking habit, alcohol consumption, perceived stress level, living with a spouse, history of disease, parity, age at menarche, menopausal status and exogenous hormone use.

^c^P value for interaction of likelihood ratio test.

^d^Parous women only with additional adjustment for age at first birth and breastfeeding.

^e^Menopausal women only.

^f^Total fertility years as the interval between menarche and menopause.

^g^*P* value for linear trend across categories of variable.

^h^Adjustment as in footnote b except for parity.

Parous women who ever breastfed versus never had a much lower risk of death by accidents [0.63 (95% CI: 0.40–0.97)] (Table 4). Insignificant positive associations were observed among women with late age at menarche [≤13 years: reference; 14–15: 1.55 (95% CI: 0.96–2.51); ≥16:1.54 (95% CI: 0.92–2.60); *P*_trend_ = 0.10] and ever use of exogenous hormones [1.45 (95% CI: 0.93–2.25)]. The effect of age at menarche was more evident in pre-menopausal women, as was that for exogenous hormone use in post-menopausal women, although neither *p*-value for interaction was significant.

**Table 4:** Hazard ratios (HRs) and 95% confidence intervals (CIs) of death by accidents according to reproductive factors for all women, pre-menopausal women, and post-menopausal women in the JPHC study.

Variable	Category	Person-years	All women					Pre-menopausal			Post-menopausal			*P*_int_^c^
			Cases	Model 1^a^		Model 2^b^		Cases	Model 2^b^		Cases	Model 2^b^		
				HR	95% CI	HR	95% CI		HR	95% CI		HR	95% CI	
Parous	No	71 289	10	1.00	ref	1.00^h^	ref	1	1.00^h^	ref	9	1.00^h^	ref	0.54
	Yes	957 293	157	1.12	0.57–2.21	1.23	0.62–2.44	31	2.02	0.27–15.3	113	1.11	0.54–2.30	
Parity^d^	1	77 207	14	1.17	0.65–2.10	1.10	0.61–1.99	5	1.45	0.52–4.03	9	0.95	0.46–1.96	0.27
	2	371 068	56	1.00	ref	1.00	ref	16	1.00	ref	40	1.00	ref	
	3	291 703	40	0.84	0.56–1.27	0.83	0.55–1.26	9	0.76	0.33–1.75	31	0.86	0.54–1.39	
	4+	217 314	47	1.15	0.73–1.80	1.13	0.72–1.77	4	0.69	0.21–2.24	43	0.74	0.75–2.01	
	*P*_trend_^g^			0.99		0.96			0.23			0.50		
Age at first birth, y^d^	≤22	213 668	40	1.00	ref	1.00	ref	6	1.00	ref	34	1.00	ref	0.54
	23–26	485 409	73	0.87	0.58–1.31	0.93	0.62–1.40	17	1.18	0.45–3.12	56	0.87	0.56–1.37	
	≥27	258 786	44	1.03	0.65–1.63	1.05	0.65–1.71	11	1.64	0.56–4.77	33	0.93	0.54–1.61	
	*P*_trend_^g^		44	0.91		0.83			0.35			0.77		
Breastfeeding^d^	Never	125 722	26	1.00	ref	1.00	ref	8	1.00	ref	18	1.00	ref	0.23
	Ever	831 570	131	0.60	0.39–0.92	0.63	0.40–0.97	26	1.15	0.43–3.05	105	0.52	0.31–0.89	
Age at menarche, y	≤13	316 896	25	1.00	ref	1.00	ref	5	1.00	ref	20	1.00	ref	0.19
	14–15	466 496	82	1.54	0.95–2.50	1.55	0.96–2.51	24	3.01	1.13–8.05	58	1.17	0.67–2.02	
	≥16	245 189	60	1.59	0.95–2.66	1.54	0.92–2.60	6	3.36	0.99–11.4	54	1.19	0.68–2.10	
	*P*_trend_^g^			0.12		0.10			0.05			0.54		
Exogenous hormone use	Never use	887 175	140	1.00	ref	1.00	ref	31	1.00	ref	109	1.00	ref	0.24
	Ever use	141 407	27	1.44	0.93–2.23	1.45	0.93–2.25	4	0.72	0.25–2.08	23	1.75	1.07–2.84	
Menopausal status	Pre menopause	481 912	25	0.96	0.58–1.59	1.04	0.63–1.73							
	Natural menopause	452 624	82	1.00	ref	1.00	ref							
	Surgical menopause	94 046	60	0.88	0.51–1.51	0.85	0.49–1.47							
Age at menopause, y^e^	≤47	177 875	42	1.00	ref	1.00	ref							
	48–50	210 330	45	0.78	0.50–1.22	0.73	0.46–1.17							
	≥51	158 485	45	0.93	0.59–1.45	0.87	0.54–1.40							
	*P*_trend_^g^			0.77		0.65								
Total fertility span, y^e,f^	≤32	204 419	51	1.00	ref	1.00	ref							
	33–35	167 339	34	0.72	0.46–1.14	0.70	0.43–1.13							
	≥36	174 911	47	0.90	0.59–1.36	0.88	0.55–1.42							
	*P*_trend_^g^	291 703		0.60		0.62								

HR, Hazard ratio; CI, Confidence interval; y, year; BMI, Body mass index.

^a^Cox proportional hazards models (using attained age as time scale stratified by 11 public health center areas)

^b^Based on model 1 and adjusted for BMI, smoking habit, alcohol consumption, perceived stress level, living with a spouse, history of disease, parity, age at menarche, menopausal status and exogenous hormone use.

^c^P value for interaction of likelihood ratio test.

^d^Parous women only with additional adjustment for age at first birth and breastfeeding.

^e^Menopausal women only.

^f^Total fertility years as the interval between menarche and menopause.

^g^*P* value for linear trend across categories of variable.

^h^Adjustments as in footnote b except for parity.

Compared with complete-case analyses, estimations derived from multiple imputations did not change substantially in terms of the magnitude or direction of the association between all reproductive factors and mortality risks of all external causes, suicide, and accidents (Supplementary Table S2). One exception was the association between accidents and breastfeeding. Regardless of increased sample size, this inverse association became null [0.75 (95% CI: 0.50–1.14)]. In the competing risk approach, the subhazard ratios for injury did not substantially differ.

## Discussion

Based on a large-scale population-based cohort study with 1 028 583 person-years, our results support the vital roles of parity and breastfeeding in the risk of all-cause and major causes of external deaths. Age at menarche and exogenous hormone use were associated with death by all injuries or accidents. Age at menarche and exogenous hormone use were also potential makers for injury. Suicide and accidents accounted for 45% and 51% of external causes of death, respectively, and thus estimations of all external causes were similar to those for suicide or accidents. Our data also revealed null associations between mortality risk by external causes and several reproductive factors, including age at first birth, menopausal status, age at menopause, and years of fertility.

### Parity

The decreased risk of suicide in women with ever parity regardless of marital status is consistent with previous studies^9,12^. A negative influence of being single (never married, separated, divorced, or widowed) is commonly quoted as a risk factor for suicide, but its impact is not much stronger in women than men^39,42^. Parenthood has an essential role in protecting from suicide^4^, and this effect may be much stronger when children are young^7^. Motherhood itself may contribute to protecting against suicide in women by inculcating a feeling of responsibility and self-worth, enhancing the social network and providing a positive social role^43^. The presence of a child may play a significant role in the decision not to commit suicide, especially while the child is dependent^8^. Older women tend to count on their adult children more than their spouse for help in difficulties with daily life^44^. As the main reason for suicide among Japanese women is physical and mental illness^45^, an adult child may confer significant emotional and material support for parents in late life.

In this study, three births were associated with the lowest risk of suicide among parous women. In contrast, previous studies suggested an association between risk of suicide and increasing parity^8^, high parity^11^ or no clear pattern^12^. The lack of a linear trend in this study may be due to the adverse effect of a large family. This possibly imposes excessive burden from physical and mental stress and economic strain on parents^14,46^. A selection effect might also explain the association between parity and suicide^15^. Women who are single because they never married or were divorced or widowed might have been aggregated to the never/low parity group. A poor health status that prevents women from becoming pregnant or completing a pregnancy, or psychiatric illness, may influence the decision to marry and have more children^47^.

### Breastfeeding

Ever breastfeeding was inversely associated with mortality risk from all external causes and accidents. In particular, a much lower risk of accidents was seen among postmenopausal women. Because no previous literature has investigated the association between breastfeeding and external causes of death, no explanation for these associations is available. One possible pathway is the protective effect of breastfeeding on several diseases after menopause including cancer, hypertension, diabetes, hyperlipidemia, and cardiovascular disease^48,49^. These are likely to increase the risk of external cause of death^35,40,50^. Another possible explanation is the protective effect of breastfeeding on osteoporosis and subsequent fracture occurrence^51,52^, and Alzheimer disease (AD)^53^, albeit that these associations remain inconclusive. However, because the association became insignificant after imputations regardless of increased sample size, this finding should be interpreted with care. The lack of detailed information on breastfeeding, such as frequency and duration, require further investigation to confirm this intriguing association between breastfeeding and the risk of death by accidents.

### Menstruation and exogenous hormone use

The marginal positive associations we saw between late age at menarche and risk of all injuries and accidents may be explained by risk of cognitive impairment^54–57^ or osteoporosis^56,57^ in later life. The delayed initiation of secretion of gonadal sex steroid influences musculoskeletal function^57,58^. Estrogen plays a positive role in regulating neuronal biochemistry and cognitive function^59^. However, evidence from epidemiological studies on the association between early and/or long exposure to estrogen and cognitive function remains inconclusive^54,55,60^.

Positive associations between external cause of death and women with ever use of OCs has been reported^13,17,18^. In contrast, controversy remains with regard to hormone therapy use^61,62^. Nevertheless, despite a potential link between exogenous hormone use and risk of injuries, earlier studies did not provide potential biological mechanisms through which exogenous hormone use might modulate the risk of injuries. Although we also showed a marginally increased risk of accidents among ever users of exogenous hormones, a lack of data limited our ability to assess the effects of OCs and HT separately. Furthermore, differences in exogenous hormone availability and formulation may prevent comparison across studies.

Our study found null associations for age at menopause, fertility years and menopausal status with risk of suicide. As the perimenopause period carries a particular risk for developing depression and higher suicidal behaviors^19^, we expected a high risk of suicide among premenopausal women. In fact, suicide is the second cause of death among Japanese women aged 30–49 years old, and its rank decreases as age category rises^63^. Interactions between hormonal change and several stressful life events such as interpersonal problems and empty nest experience may lead women to be susceptible to mental illness and subsequent suicide behaviors^24,64^.

Importantly, suicide and accidents are never the consequence of a single cause; a combination of personal, cultural, social and biological features likely interact with fluctuations in sex hormones among women^21,65,66^. Although most of the reproductive factors are not modifiable, these factors are common exposures in women. A better understanding of how reproductive history influences long-term health may help to reduce avoidable deaths by self-harm and accidents.

### Strengths and methodological issues

To our knowledge, this is the first large-scale prospective study to examine the impact of reproductive factors and the risk of external cause of death among Japanese. Strengths of the study include its large population-based sample with long follow-up period, prospective design, high response rate (more than 80%) and low loss to follow-up. The availability of a variety of reproductive factors enabled a comprehensive assessment of the relationship between reproductive factors and external causes of death. Study participants consisted of a general population across Japan, making our findings generalizable to all middle-aged Japanese women. The potential of competing risks was not particularly high in our study. In addition, external cause of death usually occurs at younger ages than most other causes of death, and our study subjects were no exception.

Several limitations should also be mentioned. First, the main findings were obtained based on complete case analyses under the assumption of missing at random, which may have introduced selection bias. However, we addressed this issue by using the imputation approach. Second, single assessment only at the baseline survey may have resulted in misclassification. However, reproductive factors of post-menopausal women did not alter, apart from the experience of exogenous hormone use. Third, although we accounted for relevant covariates, we might have failed to obtain data for other possible confounders, such as psychiatric history, genetic and familial confounding, and socio-economic status. Fourth, specific details of breastfeeding (i.e., duration and frequency) and use of any exogenous hormone (i.e., formulation, dosage, and duration) were not available. Fifth, because distributions of reproductive factors varied across generations and cultural settings, our findings might not apply to women born at different times or in other populations around the world. Finally, injuries from huge disasters may have occurred regardless of potential risk factors. However we identified that no external cause of deaths occurred among study subjects in areas which experienced a major earthquake in 2011.

## Conclusions

In summary, a lower risk of suicide was associated with ever parity and three births among parous women. Parous women who ever breastfed had a reduced risk of death by accidents. Given the few studies reported to date, our present results indicate the need for further studies to clarify the association between reproductive factors and the risk of external causes of death.

## Supplementary information

Supporting information

## Data Availability

The datasets analyzed during the current study are not available due to no permission from the ethical board.
