# Supplementary material for: Female reproductive factors and risk of external causes of death among women: The Japan Public Health Center-based Prospective Study (JPHC Study)
Source: Sci Rep. 2019 Oct 4;9:14329. doi: 10.1038/s41598-019-50890-x (PMC6778214; doi:10.1038/s41598-019-50890-x)
Supplement: Supplementary file 1 — Supporting information [file 41598_2019_50890_MOESM1_ESM.docx]

**Supporting Information**

**Female reproductive factors and risk of external causes of death among women:** **The Japan Public Health Center-based Prospective Study (JPHC Study)**

Shiori Tanaka^1,2^, Sarah K. Abe^1^, Norie Sawada^1^, Taiki Yamaji^1^, Taichi Shimazu^1^, Atsushi Goto^1^, Motoki Iwasaki^1^, Hiroyasu Iso^3^, Tetsuya Mizoue^4^, Manami Inoue^1^*, and Shoichiro Tsugane^1^

1. Epidemiology and Prevention Group, Center for Public Health Sciences, National Cancer Center, 5-1-1 Tsukiji, Chuo-ku, Tokyo 104-0045, Japan
2. Department of Global Health Policy, Graduate School of Medicine, The University of Tokyo, 7-3-1 Hongo, Bunkyo-ku, Tokyo 113-0033, Japan
3. Public Health, Department of Social Medicine, Osaka University Graduate School of Medicine, 2-2 Yamadaoka, Suita, Osaka 565-0871, Japan
4. Department of Epidemiology and Prevention, Center for Clinical Sciences, National Centre for Global Health and Medicine, 1-21-1 Toyama, Shinjuku-ku, Tokyo 162-8655, Japan

*Corresponding author:

Manami Inoue, MD, PhD

Division of Prevention, Center for Public Health Sciences, National Cancer Center

5-1-1 Tsukiji, Chuo-ku, Tokyo 104-0045, Japan

E-mail: mnminoue@ncc.go.jp

Telephone: +81 3 3542 2511 Fax: +81 3 3547 8578


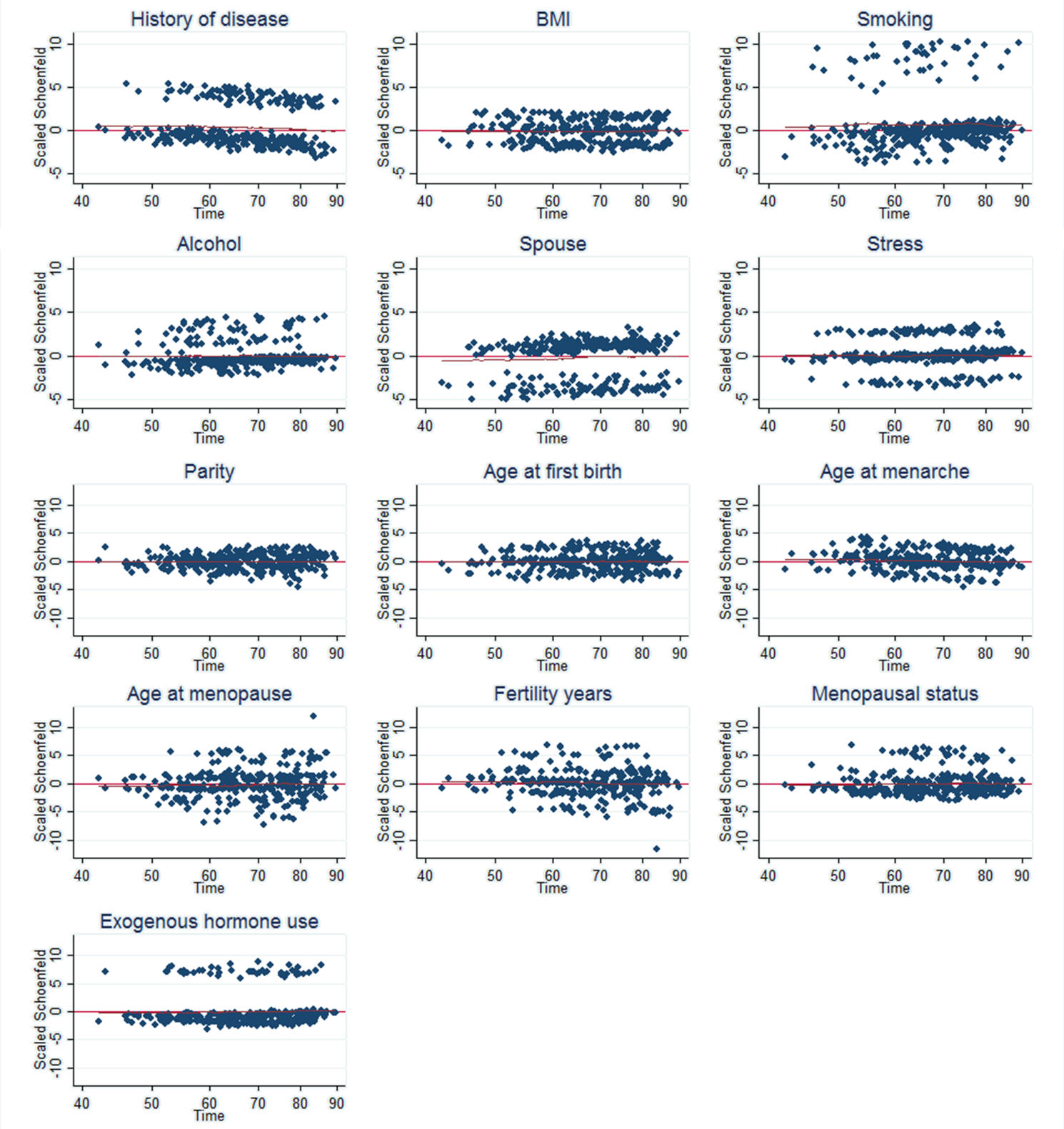


Figure S1. Results of Schoenfeld residuals in the test for proportional assumption for each variable: Plots for scaled Schoenfeld residuals and red lines for smoothed slopes with the reference line at y=0

Table S1. Basic characteristics of study subjects with and without missing data for relevant covariates at baseline survey in the JPHC study

| Characteristic | | Eligible subjects | | Subjects with complete data | | Subjects with a missing value | | *P*^a^ | |  |
| --- | --- | --- | --- | --- | --- | --- | --- | --- | --- | --- |
|  |  |  |  |  |  |  |  |  |  |  |
| Number of subjects (n) | | 60 002 | | 49 279 (82.1%) | | 10 723 (17.9%) | |  | |  |
|  | Age at recruitment, y^b^ | | 51.6 (8.0) | | 50.9 (7.8) | | 54.7 (8.1) | | <0.01 | |
|  | BMI (Kg/m^2^) ^b^ | | 23.4 (3.3) | | 23.3 (3.2) | | 23.6 (4.1) | | 0.01 | |
|  | Never smoker (%) | | 90.50 | | 90.3 | | 91.1 | | <0.01 | |
|  | Non-drinker (%) | | 75.40 | | 74.2 | | 81.2 | | <0.01 | |
|  | High perceived stress (%) | | 19.10 | | 16.5 | | 19.1 | | <0.01 | |
|  | Living with spouse (%) | | 78.30 | | 79.2 | | 74.1 | | <0.01 | |
|  | Past history of diseases (%) | | 19.90 | | 19.2 | | 23.1 | | <0.01 | |
| Reproductive factors | |  | |  | |  | |  | |  |
|  | Parity^b^ | | 2.7 (1.5) | | 2.6 (1.5) | | 2.9 (1.8) | | <0.01 | |
|  | Age at first birth, y^b,c^ | | 25.0 (3.5) | | 25.0 (3.5) | | 24.7 (3.6) | | <0.01 | |
|  | Ever breastfed (%)^c^ | | 86.8 | | 86.8 | | 86.6 | | 0.67 | |
|  | Age at menarche, y^b^ | | 14.6 (1.9) | | 14.5 (1.8) | | 15.4 (2.2) | | <0.01 | |
|  | Age at menopause, y^b,d^ | | 48.1 | | 48.1 (4.8) | | 47.9 (5.5) | | 0.01 | |
|  | Total fertility years^b,d,e^ | | 32.8 | | 33.0 (4.8) | | 32.1 (5.4) | | <0.01 | |
|  | Ever use of exogenous hormone (%) | | 13.2 | | 13.4 | | 12.2 | | <0.01 | |

BMI, Body mass index; y, year

^a^ Analysis of variance (ANOVA for continuous variables or chi-square test for categorical variables)

^b^ Mean (standard deviation)

^c^ Parous women only

^d^ Post menopause only

^e^ Total fertility years as the interval between menarche and menopause

Table S2. Hazard ratios (HRs) and 95% confidence intervals (CIs) of death by all external causes, suicide, and accidents associated with reproductive factors using multiple imputed datasets in the JPHC study^a^

| Variable | Category | Person-years | All external causes | | | Suicide | | | Accidents | | | | |
| --- | --- | --- | --- | --- | --- | --- | --- | --- | --- | --- | --- | --- | --- |
|  |  |  | Cases | HR | 95% CI | Cases | HR | 95% CI | Cases | HR | | 95% CI | |
| Parous | No | 85 453 | 35 | 1.00^f^ | ref | 20 | 1.00^f^ | ref | 12 | | 1.00^f^ | | ref |
|  | Yes | 1 161 489 | 410 | 0.76 | 0.55–1.10 | 174 | 0.60 | 0.38–0.95 | 193 | | 1.14 | | 0.64–2.01 |
| Parity^b^ | 1 | 94 819 | 40 | 1.04 | 0.72–1.51 | 20 | 1.12 | 0.64–1.94 | 15 | | 0.88 | | 0.50–1.56 |
|  | 2 | 440 845 | 145 | 1.00 | ref | 72 | 1.00 | ref | 67 | | 1.00 | | ref |
|  | 3 | 351 076 | 128 | 0.79 | 0.60–1.03 | 50 | 0.66 | 0.42–1.01 | 65 | | 0.89 | | 0.61–1.31 |
|  | 4+ | 276 870 | 97 | 0.98 | 0.72–1.33 | 32 | 0.53 | 0.53–1.41 | 56 | | 1.10 | | 0.73–1.66 |
|  | *P*_trend_^e^ |  | 35 | 0.51 |  |  | 0.20 |  |  | | 0.56 | |  |
| Age at first birth^b^ | ≤22 | 264 701 | 86 | 1.00 | ref | 38 | 1.00 | ref | 43 | | 1.00 | | ref |
|  | 23-26 | 587 544 | 222 | 0.99 | 0.75–1.20 | 95 | 0.93 | 0.62–1.39 | 109 | | 1.05 | | 0.72–1.51 |
|  | ≥27 | 309 245 | 102 | 1.10 | 0.80–1.50 | 41 | 0.90 | 0.57–1.42 | 50 | | 1.22 | | 0.79–1.89 |
|  | *P*_trend_^e^ |  | 35 | 0.55 |  |  | 0.65 |  |  | | 0.34 | |  |
| Breastfeeding^b^ | Never | 151 844 | 69 | 1.00 | ref | 28 | 1.00 | ref | 35 | | 1.00 | | ref |
|  | Ever | 1 009 647 | 341 | 0.70 | 0.53–0.93 | 146 | 0.73 | 0.47–1.16 | 167 | | 0.75 | | 0.50–1.14 |
| Age at menarche, years | ≤13 | 358 704 | 78 | 1.00 | ref | 39 | 1.00 | ref |  | | 1.00 | | ref |
|  | 14-15 | 556 235 | 227 | 1.14 | 1.05–1.78 | 106 | 1.52 | 1.03–2.22 | 33 | | 1.19 | | 0.80–1.78 |
|  | ≥16 | 332 005 | 140 | 1.17 | 0.86–1.59 | 49 | 1.22 | 0.77–1.94 | 100 | | 1.14 | | 0.74–1.76 |
|  | *P*_trend_^e^ |  | 35 | 0.31 |  |  | 0.39 |  | 81 | | 0.55 | |  |
| Exogenous hormone use | Never use | 1 077 325 | 391 | 1.00 | ref | 172 | 1.00 | ref | 185 | | 1.00 | | ref |
|  | Ever use | 169 619 | 54 | 1.03 | 0.75–1.42 | 22 | 0.85 | 0.54–1.36 | 29 | | 1.24 | | 0.81–1.89 |
| Menopausal status | Pre menopause | 481 912 | 113 | 0.90 | 0.67–1.19 | 72 | 1.08 | 0.70–1.68 | 36 | | 0.92 | | 0.58–1.46 |
|  | Natural menopause | 452 624 | 287 | 1.00 | ref | 105 | 1.00 | ref | 155 | | 1.00 | | ref |
|  | Surgical menopause | 94 046 | 43 | 0.88 | 0.63–1.23 | 17 | 0.99 | 0.56–1.77 | 22 | | 0.93 | | 0.59–1.46 |
| Age at menopause, years^c^ | ≤47 | 234 944 | 99 | 1.00 | ref | 32 | 1.00 | ref |  | | 1.00 | | ref |
|  | 48-50 | 279 204 | 142 | 0.94 | 0.70–1.26 | 65 | 1.14 | 0.68–1.59 | 55 | | 0.78 | | 0.52–1.16 |
|  | ≥51 | 202 936 | 89 | 0.87 | 0.64–1.19 | 27 | 0.82 | 0.48–1.38 | 69 | | 0.87 | | 0.57–1.32 |
|  | *P*_trend_^e^ |  | 102 | 0.38 |  |  | 0.55 |  | 53 | | 0.56 | |  |
| Total fertility span, years^c, d^ | ≤32 | 278 611 | 95 | 1.00 | ref | 38 | 1.00 | ref | 55 | | 1.00 | | ref |
|  | 33-35 | 213 780 | 160 | 0.87 | 0.64–1.17 | 51 | 1.06 | 0.65–1.73 | 77 | | 0.72 | | 0.48–1.07 |
|  | ≥36 | 218 693 | 75 | 0.95 | 0.67–1.35 | 35 | 0.93 | 0.58–1.50 | 45 | | 0.97 | | 0.60–1.56 |
|  | *P*_trend_^e^ |  | 78 | 0.75 |  |  | 0.81 |  |  | | 0.83 | |  |

HR, Hazard ratio; CI, Confidence interval; y, year; BMI, Body mass index

^a^ Cox proportional hazards models using attained age as time scale stratified by 11 public health center areas and adjusted for BMI, smoking habit, alcohol consumption, perceived stress level, living with a spouse, history of disease, parity, age at menarche, menopausal status and exogenous hormone use using multiple imputed datasets. Multiple imputations by the chained equations approach with 20 iterations were performed to impute missing values by including all covariates, person-years, and vital status. Estimations were then combined using Rubin’s rules (the STATA mi procedure). Estimations were restricted to parous women for breastfeeding and age at first birth, and to postmenopausal women for age at menopause and total fertility years.

^b^ Parous women only with additional adjustment for age at first birth and breastfeeding

^c^ Menopausal women only

^d^ Total fertility years as the interval between menarche and menopause

^e^ *P* value for linear trend across categories of variable

^f^ Adjustments as in footnote except for parity
